# Amino Acid Distribution Rules Predict Protein Fold: Protein Grammar for Beta-Strand Sandwich-Like Structures

**DOI:** 10.3390/biom5010041

**Published:** 2015-01-23

**Authors:** Alexander Kister

**Affiliations:** Department of Mathematics, Rutgers University, Piscataway, NJ 08854, USA; E-Mail: akister@math.rutgers.edu

**Keywords:** sequence–structure relationship, protein fold, supersecondary structure, structure prediction

## Abstract

We present an alternative approach to protein 3D folding prediction based on determination of rules that specify distribution of “favorable” residues, that are mainly responsible for a given fold formation, and “unfavorable” residues, that are incompatible with that fold, in polypeptide sequences. The process of determining favorable and unfavorable residues is iterative. The starting assumptions are based on the general principles of protein structure formation as well as structural features peculiar to a protein fold under investigation. The initial assumptions are tested one-by-one for a set of all known proteins with a given structure. The assumption is accepted as a “rule of amino acid distribution” for the protein fold if it holds true for all, or near all, structures. If the assumption is not accepted as a rule, it can be modified to better fit the data and then tested again in the next step of the iterative search algorithm, or rejected. We determined the set of amino acid distribution rules for a large group of beta sandwich-like proteins characterized by a specific arrangement of strands in two beta sheets. It was shown that this set of rules is highly sensitive (~90%) and very specific (~99%) for identifying sequences of proteins with specified beta sandwich fold structure. The advantage of the proposed approach is that it does not require that query proteins have a high degree of homology to proteins with known structure. So long as the query protein satisfies residue distribution rules, it can be confidently assigned to its respective protein fold. Another advantage of our approach is that it allows for a better understanding of which residues play an essential role in protein fold formation. It may, therefore, facilitate rational protein engineering design.

## 1. Introduction

### 1.1. Computer Methods for Analysis of Sequence–Tertiary Structure Relationship

Anfinsen’s discovery [1] that “the native conformation [of a protein] is determined by the totality of interatomic interactions and hence by the amino acid sequence, in a given environment”, makes it possible, in theory, to deduce tertiary structure from a primary sequence. The most direct way to do so is via *ab initio* protein calculation approach, which is based on analysis of the energy landscape that results from residue–residue interactions [2,3,4]. The advantage of *ab initio* approach is that it only takes into account the physicochemical properties of residues in a sequence and does not require prior knowledge of known structures. It also affords insight into relative contributions of residues to structure formation [5,6]. The disadvantage of this approach is the necessity of carrying out energy calculations to a high degree of accuracy, of a very large number of different strong and weak interactions–electrostatic contacts, hydrogen bonds, van der Waals and hydrophobic interactions [7,8,9,10]. Calculations of water-mediated hydrophobic interactions, a dominant driving force of the folding process, present an especially difficult challenge [11,12]. Moreover, energy calculations require a high degree of precision as folded conformation is generally only 5–10 kcal/mol more stable than the unfolded chain [13]. Currently, the application of *ab initio* structure modeling is limited because of the computational difficulties [14,15].

The other principal approach to sequence–structure prediction is based on the famous conclusion by Chothia and Lesk that “the extent of the structural changes is directly related to the extent of the sequence changes” [16]. In fact, if a query protein shows detectable homology to a sequence of a protein with known three-dimensional structure, usually more than 25%, then the two proteins likely have a similar tertiary structure. Thus, a fairly accurate model of 3D structure can usually be obtained via homology modelling [17,18]. Currently, various template-based structural modeling methods are the most widely and successfully used techniques for protein structure prediction [19].

The most important limitation of template-based methods for structure prediction is that they cannot be applied unless there exists a homologous protein whose 3D structure has already been deciphered. Presently, there is a huge gap between the number of sequenced amino acid chains and X-ray deciphered protein structures and many query sequences do not have homologues with known structure.

Another difficulty with the homology approach is the uncertainty of determining the extent of the sequence changes. In fact, the percentage of dissimilar residues in proteins with similar structures may be surprisingly large—greater than 75%. A possible explanation for structural similarity of proteins with widely divergent residue content is that a subset of residues in a sequence at certain specified positions largely defines the 3D structure of a protein, while the other residues have a minor effect on the fold. The idea that relative contributions of residues to structure stability may be very different is widely accepted in protein science [20,21,22]. This conclusion has been corroborated by numerous mutagenesis experiments, which revealed that substitutions of different residues at various positions in sequences have a quite variable effect on structure and stability of proteins [23,24,25].

The assumption that all residues in a sequence can be classified as either “favorable” residues, that are mainly responsible for a given fold formation, or “supportive residues”, that help maintain stability of the structure, allows us to explain two seemingly contradictory observations. First, it may explain why proteins with very high sequence identity may have very different structures [26]. For example, two natural proteins with 88% sequence identity were found to have very different tertiary structures: a 3-alpha helix fold and an alpha/beta fold [27]. It is plausible to suggest that if 12% of dissimilar residues include most of the “favorable” residues, then the two sequences will fold differently despite the high degree of overall homology.

The inverse has also been observed: different protein families may have the same tertiary fold despite very low sequence similarity. This is the case, for example, for proteins of 15 distinct enzyme families with the same TIM-barrel fold [28,29]. Here, similar residues in sequences are the favorable residues and they are mainly responsible for protein structure formation. Neither of these two examples contradict Chothia–Lesk’s rule so long as we assess the “extent of sequence change” as “change of favorable residues” only. Then, proteins with the same fold may share the favorable residues, while the majority of supporting residues would be very different. Conversely, in proteins with unlike structures, supportive residues may show a high degree of similarity, yet, because the favorable residues are different, the sequences will encode dissimilar structures.

In summary, both *ab initio* and homology methods for sequence–structure predictions have significant limitations, and it is important to develop alternative approaches that would overcome some of these limitations.

### 1.2. Alternative Approach for Analysis of Sequence–Structure Relationship

The proposed approach is based on the premise that distribution of a relatively small number of favorable residues within a sequence largely defines the polypeptide fold. Another set of residues—“unfavorable” residues—are incompatible with that fold. A corollary of this hypothesis is that the list of favorable residues with specified allowed distances between them, and the list of unfavorable residues with specified locations in a sequence, constitute the defining sequence characteristics of a particular fold. A query sequence can then be tested against a list of favorable and unfavorable residues to determine whether it belongs to the particular fold or not.

### 1.3. Outline of the Approach to Determining Favorable and Unfavorable Residues of a Fold

Step 1: Selection of a representative set of proteins with a specified fold from 3D structure databases.

Step 2: Identification of secondary structures and supersecondary structure (arrangement of strands and/or helices in space) in proteins with specified fold.

Step 3: Identification of strands and/or helices that are crucial for the proper folding of the polypeptide chain—“the structural invariant” of the fold.

Step 4: Assumptions about favorable and unfavorable residues both within the structural invariant and in other fragments of sequence based on general principles of protein structure formation and structural features specific to a given protein fold.

Step 5: Verification of the initial assumptions for the given set of proteins; refining the initial assumptions of favorable and unfavorable residues–iterative steps. Testing sensitivity of resulting assumptions about favorable and unfavorable residues (“rule set”) for identifying proteins with the specified fold.

Step 6: Testing specificity of the rule set determined in Step 5 on all other proteins.

Borrowing an analogy from linguistics, we can say that our approach involves both morphologic analysis (Steps 4 and 5) and syntactic analysis (Step 2). Morphology is primary concerned with the internal structures of words, which, in protein “language” correspond to secondary structure units-strands, helices, and loops. Syntax is primary concerned with the ways in which words are put together—the spatial interrelationship between strands and helices in proteins, the supersecondary structure.

**Figure 1:**
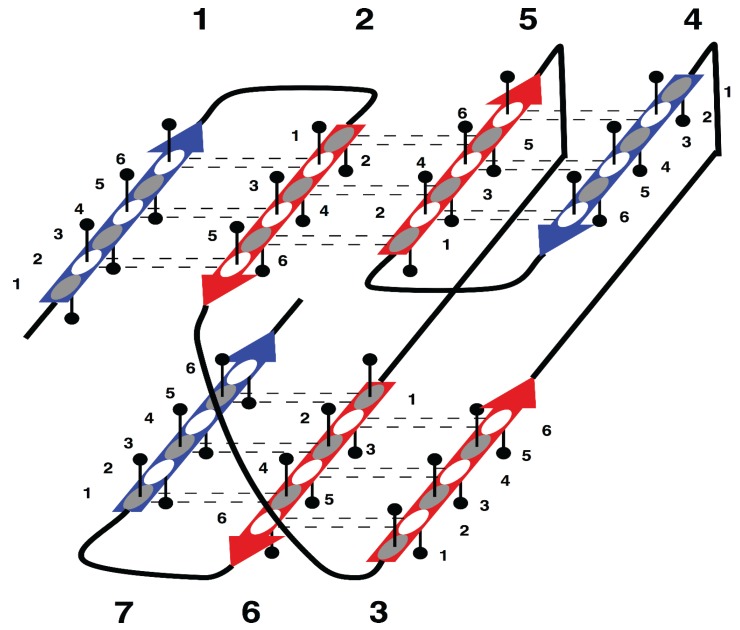
Supersecondary structure of sandwich-like proteins with a specific “interlock” arrangement of strands 2, 3, 5 and 6. Beta strands are represented by arrows and protein loops are shown as lines. The interlock strands are shown in red. Six positions in each strand are considered. In this model, it is assumed that residues at positions 1, 3 and 5 are directed inside (hydrophobic positions), whereas residues at positions 2, 4 and 6 are on the surface (hydrophilic positions).

The specific aim of our study is the analysis of morphology and syntax in a group of beta proteins with seven beta strands. The arrangement of the strands in the two beta sheets, which form the so-called sandwich-like structure, is shown in Figure 1.

## 2. Strategy for Determining Identity and Localization of Favorable and Unfavorable Residues

### 2.1. Selection of Beta-Sandwich-Like Proteins

All proteins with known structures from the SCOPe database [30] were divided into two groups: beta proteins with a sandwich-like structure with the fold shown in Figure 1, hereafter referred to as “target proteins”, and proteins with any other kind of folds, “other proteins”. The detailed procedure for identification of secondary structure and supersecondary structure of target proteins was described in our previous paper [31].

### 2.2. Supersecondary Structure Features Common to All Target Proteins: Structural Invariant of the Fold

Supersecondary structure (SSS) is hierarchically intermediate between secondary and tertiary structures. It was introduced by M. Rossman to emphasize the conserved combinations of secondary structure elements in 3D space [32,33]. There are several reasons for using SSS rather than the atomic structures of proteins in our study. First, SSS of a protein defines arrangement of secondary structure elements in space and has a strict and unambiguous definition. Beta-proteins with the same SSS have the same number of strands and the same arrangement of strands in beta sheets. No other structural features, such as, for example, number of residues within strands, or a conformation of loops, are taken into account when comparing SSS of proteins. Secondly, proteins with an identical SSS may belong to different families, have diverse protein functions and hence, possibly, very little global sequence similarity. Proteins with similar SSS, but widely dissimilar primary sequences, are the most informative for discovering residue distribution rules. A further advantage of SSS is that it simplifies protein modeling analysis. Use of “protein skeletons”—SSS-rather than full-bodied atomic three-dimensional protein structures—allows one to uncover the common structural features that are specific and unique to a given fold.

To facilitate analysis of SSS, we introduced the notion of SSS unit, called “strandon”, defined as a set of consecutive strands in sequences that are adjacent to each other in space and connected by hydrogen bonds in a beta-sheet [34]. An example of a strandon in Figure 1 is the sequence fragment made up of the consecutive strands 1 and 2 that are H-bonded to each other in the beta sheet. Strand 3 is not part of the first strandon, because it has no H-bonds with strand 2. Strand 3 also has no H-bonds with strand 4 and therefore it constitutes a strandon by itself. Analogous analysis shows that the other two strandons in this structure are made up of strands 4 and 5, and 6 and 7, respectively.

Analysis of all beta sandwich structures with two beta sheets (according to the SCOP classification) revealed certain constraints for localization of consecutive strands in strandons and beta sheets [34,35]. These constraints were found in 90% of such structures.

Constraint 1. If two consecutive strands in a sequence do not share hydrogen bonds within a strandon, then they cannot be in one beta sheet.

For example, consecutively numbered strands 1, 2 and 3, are not allowed to have an order of strands 1-3-2 or 2-1-3 within a beta sheet, because pairs of strands 1 and 2, or 2 and 3, would then have no hydrogen bond contact.

Constraint 2. There are always two consecutive strands at the same edge of two beta-sheets (left and right).

**Figure 2:**
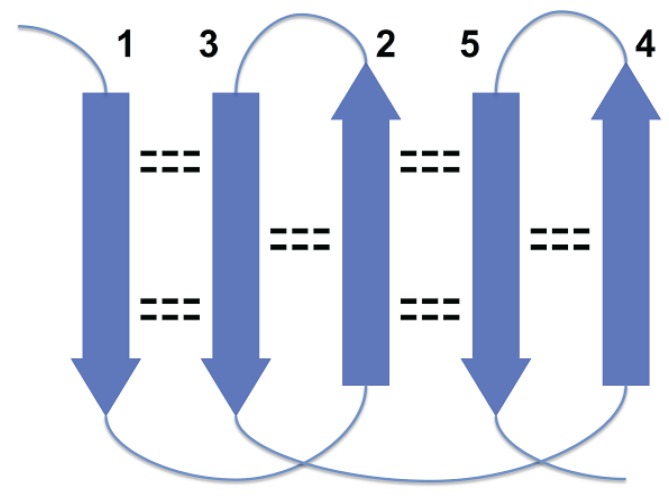
Disallowed arrangement of strands 1, 2 and 3 in a beta sheet because of loops overlapping.

In Figure 1, strands 3 and 4 are at the right edges of respective beta-sheets, and strands 1 and 7, at the left edge (strands 1 and 7 are consecutive in cyclic ordering). For the most part, these constraints are the consequence of a general structural rule for proteins–avoidance of energetically unfavorable loop overlapping (Figure 2) [36].

Constraint 3. There are always at least two pairs of consecutive strands *i*, *i* + 1 and* j*, *j* + 1 such that strand *i* has H-bond contacts with strand *j* in one beta sheet and strand* i* + 1 has a H-bond with strand *j* + 1 in the another beta sheet.

This specific configuration of the strands—*i*, *i* + 1,* j, j* + 1, which correspond to strands 2, 3, 5 and 6 in target proteins (Figure 1), was termed an “interlock”, because the two pairs of strands are “interlocked”. This four-strand configuration is found in all sandwich-like proteins, but not in any other beta structures [35]. Thus, the interlock is the structural invariant of sandwich-like beta proteins. We suppose that formation of H-bond contacts between non-consecutive strands in the interlock is due to specific distribution of favorable residues in these strands. Therefore, the search for favorable residues was focused on the interlock strands.

### 2.3. Iterative Search Algorithm for Determining Favorable and Unfavorable Residues in Strands and Loops: An Overview

The process of determining favorable residues and unfavorable residues is iterative. The starting assumptions are based on the knowledge of: (a) the main structural characteristics, which define the arrangement of strands in a supersecondary structure, such as interlock and non-interlock strands and edge strands in beta sheets; (b) the relative frequencies of each amino acid in beta strands, alpha helices and loops in proteins; (c) preferential localization of residues in strands and loops required for formation of hydrophobic core and hydrophilic surface.

These assumptions lead to testable predictions about which residues are essential for the given fold, and which are disallowed or restricted in a particular strand or loop. The list of favorable residues, together with allowed distances between them, and the list of unfavorable residues in particular strands and loops should be checked against the set of all target proteins. In this test of sensitivity, each assumption is checked separately. If the initial assumption results in a large number of false negatives, then the assumption is rejected, or restricted in the next iteration, for example, by decreasing the number of allowed favorable residues in a strand or requiring that the specific residue be present in the interlock strands only, but not in all strands. If, on the other hand, the initial assumption is found to be valid for all target proteins then it is possible that the assumption is overly permissive and it is necessary to test a more restricted version in the next iteration. An example of a restriction would be to allow Pro to occupy not any positions in strands, but only hydrophilic positions. When an iteration test for presence or absence of a specific residue in specific position, yields a high true positive rate,* i.e.*, the sequence characteristic is observed in all or almost all “target” proteins, then the given property is considered as “sensitive favorable or unfavorable” residue(s).

At the next stage, the list of all the favorable and unfavorable residues and their positions in sequences that are highly sensitive for target proteins is then subjected to test of specificity against a group of all “other proteins”. In this stage, all assumptions are tested together. If the number of the assumptions is not sufficient for the specificity analysis and the test results in a large number of false positives, a new assumption is proposed. This new assumption is first checked for its sensitivity for target proteins. If it is found in target proteins, it is then added to the extended list of assumptions, which is then subjected to test of specificity on all “other proteins”. If the list of favorable and unfavorable residues has an acceptable specificity then it is adopted as a set of rules governing distribution of favorable and unfavorable residues in the sequence of a specified fold. We argue here that these rules can serve for predicting which sequences will have the specified fold.

### 2.4. An Example of Using the Search Algorithm to Determine Rules of Distribution of Proline Residue in Target Proteins

For analysis of strands, we consider six residue positions in each of the seven strands in target proteins (Figure 1). From the geometry of strands, it can be deduced that the side chains of residues point roughly perpendicularly to the plane of the sheet in alternating directions, outward and inward, of the structure interior. We will assume that residues at even positions in strands are directed inside and form a protein’s hydrophobic interior (hydrophobic positions), while residues at odd positions form the protein surface (hydrophilic positions). “Residue content” at strand positions is analyzed first separately for hydrophobic positions and for hydrophilic positions, and then for the distribution of residues in all positions for every strand.

Loops lengths in target proteins vary from three to 16 residues and total length of the six loops, from 30–60 residues. Due to the very high variability among loop fragments, it was not possible to assign favorable and unfavorable residues to particular positions within loops.

Let us consider here the iterative steps of the search algorithm on the example of the rule limiting distribution of Proline (Pro) residue in strands. Analysis of Pro frequency in proteins shows that this residue is very rarely found in strands. Therefore, our initial assumption is that Pro is not present at any position in either interlock or non-interlock strands. This assumption is tested in all target proteins.

The testing of the assumption that seven strands of target proteins contains no Pro residues, involves the following steps: we assign the first residue in the sequence to the first position of strand 1 and the five subsequent residues to the remaining positions of strand 1. If the test reveals Pro at any position in this variant of the strand, then the starting site for the strand is shifted by one position in a sequence and the test is repeated. If the test shows no Pro in this fragment, then the definition of strand 1 is upheld at this stage.

The length of a loop between strands 1 and 2 is varied between three and 16. Starting with the shortest allowed loop of three residues, the fragment corresponding to the second strand is defined and Pro occupancy is queried in the assumed positions of strand 2. If there are no Pro at any of the six positions of presumed strand 2, then the test is applied to the next fragment that could correspond to strand 3. If the test finds Pro within putative strand 2, then this strand definition is rejected, the length of the loop between strands 1 and 2 is increased by one residue, and Pro test is applied to the newly defined strand 2. If the length of the loop is increased to maximum allowed length of 16 residues and yet all attempts to find strand 2 that would not contain a Pro fail, then the algorithm is restarted with a new definition of strand 1, which is shifted by one residue relative to the previous definition. This procedure is repeated for every strand until a strand assignment is achieved, which satisfies the rules of Pro placement. If the iteration procedure checked for all possible definition of strands in a sequence and failed to find a secondary structure with no Pro residue in any strands, then the assumption of “no Pro allowed in any strand” cannot be satisfied for the given query sequence. Our analysis shows that “no Pro in any strand” assumption is not valid for about half of target proteins. Consequently, we abandon this initial assumption as overly restrictive and replace it with a modified assumption about Pro residues that would give us a better fit with the data. Through trial-and-error approach, we are able to formulate the rules of distribution of Pro that satisfies all of target proteins (see Section 3).

## 3. Distribution of Residues in Strands and Loops of Target Proteins

### 3.1. Distribution of Residues at Hydrophobic Positions (Table 1)

Most of the rules describing specific distributions of amino acids in proteins are consequence of the requirement of a hydrophobic core in proteins. The conjecture that the hydrophobic core effect is the dominant structure-determining factor for protein stability was put forth even before the first protein structures were deciphered [37]. Numerous observations of protein structures have since confirmed this assumption [38,39,40,41,42]. For sandwich proteins with two β-sheets packed face-to-face, hydrophobic residues are located in the interior between the beta sheets and form the hydrophobic core. Because residues of the interlock strands contribute the most to structure stability, it may be assumed that there are relatively more hydrophobic residues in the interlock strands than in the non-interlock strands. This premise is the starting assumption in the iterative algorithm to determine rules of hydrophobic residue distribution in strands of target proteins. Our iterative algorithm yielded the following rules:

(1) *Rules for hydrophobic residues in strands* (Table 1, row 1):
  - Interlock strands:
    - three positions in the interlock strands 2 and 3 are occupied by hydrophobic residues;
    - at least one position in the interlock strands 5 and 6 is occupied by a hydrophobic residue;
    - the total number of hydrophobic residues in strands 5 and 6 is between three and six residues.
  - Non-interlock strands:
    - at least one position in strands 1, 4 and 7 is occupied by a hydrophobic residues;
    - the total number of hydrophobic residues in these strands is between four and six residues.
  - All strands:
    - no more than five of the 21 hydrophobic positions may be occupied by non-hydrophobic residues.
(2) *The rules for a subset of* *hydrophobic residues* *Ile, Trp, Leu, Phe, Met and Val* (Table 1, row 2):
  Hydrophobic residues Ala and Cys were excluded from this analysis because of the small accessible surface, and Tyr was excluded because it is a “partially” hydrophobic residue with a polar side group. The analysis is thus restricted to the residues with strong hydrophobic interactions in strands.
  - Interlock strands:
    - at least, one residue from this group (Ile, Trp, Leu, Phe, Met and Val) is present in each of the interlock strands;
    - the total number of the hydrophobic residues in these strands is between six and 10 residues.
  - Non-interlock strands:
    - the presence of residues Ile, Trp, Leu, Phe, Met and Val is not mandatory in any one of the non-interlock strands, but no less than two and no more than five of these residues must be present in these strands.
(3) *The rule for residue Ala* (Table 1, row 3)
  For some of the hydrophobic residues, the frequency of occurrence in the hydrophobic core is relatively small. This may be due to hydrophobic indices and structural features of these residues. Residue Ala, because it is the smallest in volume among the hydrophobic residues, cannot form strong hydrophobic interaction. Our analysis showed strict restrictions on Ala placement in hydrophobic interior:
  - no more than one Ala is allowed in any of the strands;
  - no more than two Ala are allowed in all of the strands.
(4) *The rule for residue Cys* (Table 1, row 4)
  Residue Cys has a polar thiol group and can be classified as a hydrophilic amino acid. However, Cis are often located in hydrophobic part of proteins, and therefore Cys is usually classified with the hydrophobic amino acids. The role of Cys residues for stability of beta sandwich structures is paramount in many proteins in which two Cys are located in two different sheets and form a “disulfide bridge”. Our analysis shows that:
  - no more than one Cys is allowed within any one strand;
  - no more than three Cys in total are allowed in all of the strands.
(5) *The rule for residue Tyr* (Table 1, row 5):
  Residue Tyr has a polar side chain hydroxyl group, but it is usual to observe Tyr substitution for aromatic hydrophobic residues. Tyr is considered here as a partially hydrophobic amino acid with limited participation in hydrophobic core formation. Analysis shows that:
  - no more than one Tyr residue is allowed in any one strand;
  - no more than two Tyr in total are allowed in all of the strands.
(6) *The rule for Aromatic residues* (Table 1, row 6):
  Aromatic residues Phe, Trp and Tyr are less commonly observed than aliphatic hydrophobic residues in the hydrophobic core, probably because of their large volumes. It has long been known that protein interior is closely packed [43]. Perhaps, this structural compactness of the hydrophobic core is best carried out by aliphatic amino acids with a smaller volume of side chains. The rules pertaining to aromatic residues reflect this reality:
  - no more than two aromatic residues are allowed in any one interlock strand;
  - no more than three aromatic residues in total are allowed in the interlock strands;
  - for non-interlock strands, only one aromatic residue is allowed;
  - in total, no more than three aromatic residues are permitted within strands of a beta sandwich structure.
(7) *The rules for non-hydrophobic residues in hydrophobic positions* 
  The analysis of hydrophobic positions (Table 1, row 1) showed that they are occupied by no more than 17 hydrophobic residues, and,
  - at least, four non-hydrophobic residues can be found among the 21 hydrophobic positions in the seven strands.
  Specific rules governing distribution of non-hydrophobic residues are as follows:
(8) *The rules for residues Pro and Gly* 
  Pro and Gly are considered “structure disruptors” and have low-frequency in strands.
  ii. no Pro residue is allowed in any interlock strand (Table 1, row 7);
  iii. only one Pro is allowed in non-interlock strands;
  iv. no Gly residue is allowed in any of the strands (Table 1, row 8).
(9) *The rules for charged residues* (Table 1, row 9)
  Charged amino acids have favorable contact with water, and only a limited number of these residues may be found in a protein’s hydrophobic interior. However, the number of charged residues may be greater than the number of aromatic hydrophobic residues.
  v. no charged residues are allowed in interlock strands 2 and 3;
  vi. no more than two charged residues are allowed in interlock strands 5 and 6;
  vii. no more than three charged residues are allowed in non-interlock strands 1, 3 and 6;
  viii. no more than four charged residues are allowed in total in all of the strands.

**Table 1:** The rules of assignment of residues at hydrophobic positions in strands.

Rule No.	Residues	Interlock Strands				Total	Non-Interlock Strands			Total	Total
		Strand 2	Strand 3	Strand 5	Strand 6	Interlock Strands	Strand 1	Strand 4	Strand 7	Non‑Interlock Strands	All Strands
1	Trp, Ile, Phe, Leu, Cys, Val, Met, Ala, Tyr	3	3	≥1	≥1	≥9 and ≤12	≥1	≥1	≥1	≥4 and ≤6	≥13 and 17≤
2	Trp, Ile, Phe, Leu, Val, Met	≥1	≥1	≥1	≥1	≥6 and ≤10	≥0	≥0	≥0	≥2 and ≤5	≥8 and 15≤
3	Ala	1≤	1≤	1≤	1≤	2≤	1≤	1≤	1≤	2≤	2≤
4	Cys	1≤	1≤	1≤	1≤	2≤	1≤	1≤	1≤	2≤	2≤
5	Tyr	1≤	1≤	1≤	1≤	1≤	1≤	1≤	1≤	1≤	2≤
6	Phe, Trp, Tyr	2≤	2≤	2≤	2≤	3≤	1≤	1≤	1≤	1≤	3≤
7	Pro	0	0	0	0	0	1≤	1≤	1≤	1≤	1≤
8	Gly	0	0	0	0	0	0	0	0	0	0
9	Asp, Glu, Arg, Lys, His	0	0	2≤	2≤	2≤	2≤	2≤	2≤	3≤	4≤

**Table 2:** The rules of assignment of residues at hydrophilic positions in strands.

Rule No.	Residues	Interlock Strands				Total	Non-Interlock Strands			Total	Total
		Strand 2	Strand 3	Strand 5	Strand 6	Interlock Strands	Strand 1	Strand 4	Strand 7	Non-Interlock Strands	All Strands
1	Gln, Glu, Arg, Thr, Ser, Tyr, Asp, His, Lys, Asn	≥1	≥1	≥1	≥1	≥7 and 10≤	≥1	≥1	≥1	≥5 and 8≤	≥13 and 18≤
2	Glu, Arg, Lys, Asp, His	≥0	≥0	≥0	≥0	≥0 and 5≤	≥0	≥0	≥0	≥0 and 6≤	≥1 and 9≤
3	Pro	0	0	1≤	0	1≤	1≤	1≤	1≤	2≤	2≤
4	Gly	1≤	1≤	1≤	1≤	2≤	1≤	1≤	1≤	1≤	1≤
5	Pro + Gly	1≤	1≤	1≤	1≤	3≤	1≤	1≤	1≤	2≤	3≤

### 3.2. Distribution of Residues at Hydrophilic Positions (Table 2)

(1) *The rule for hydrophilic residues* (Table 2, row 1)
  - at least one position in any strand is occupied by a hydrophilic residue. In total, there are at least 13 hydrophilic residues in all beta strands.
  Comment: the total numbers of hydrophobic residues at the positions designated as “hydrophobic” and of hydrophilic residues at the “hydrophilic” positions are approximately the same in all strands.
(2) *The rule for charged residues* (Table 2, row 2)
  No specific rules of distribution of charged residues were discovered. There are structures where these residues are not found at all. However, there was an upper limit of 9 on the number of charged residues was found.
(3) *The rules for Pro and Gly residues:* 
  - Pro is not allowed in interlock strands 2, 3 and 5. In the other strands, no more than one Pro is allowed (Table 2, row 3);
  - maximum number of Pro in all of the strands is two;
  - no more than one Gly is allowed in any strand (Table 2, row 4);
  - maximum number of Gly in all of the strands is two;
  - no combination of Pro and Gly is allowed in any strand (Table 2, row 5);
  - the total number of Gly and Pro residues in all strands is no more than three.

### 3.3. Distribution of Residues within Strands

The rules about favorable and unfavorable residue content within strands without regard to their position are presented in Table 3.

### 3.4. Distribution of Residues within Loops

The criteria for selecting a sequence fragment to be a loop in a 3D structure are based on prior statistical analysis of residue content in loops of proteins [44,45]. To formalize the approach to loop definition, we introduce the notion of “loop-favorable function”.

(1) *The rule for a loop-favorable function* 
  The most favorable residues in loops, Gly and Pro, were assigned value of 2, while the other loop-favorable residues—Asp, Asn, His, Ser and Thr—were assigned a value of 1. All other residues have a value of 0. The sum of these values in a putative loop fragment is the value of the “loop favorable function”. The following criterion is used to identify a sequence fragment compatible with a loop; the rules for the loop favorable function: the value of the loop favorable function must be at least twice as large as the number of residues in a loop.
(2) *The rule for hydrophobic residues* 
  Analysis of frequencies of hydrophobic residues (Trp, Ile, Phe, Leu, Val and Met) within loops with that of other residues yields additional rules for loop selection:
  - a loop must contain less hydrophobic residues than hydrophilic residues;
  - there are to be no more than 12 hydrophobic residues in all five loops.

**Table 3:** The rules of assignment of residues at any position in strands.

Rule No.	Residues	Interlock Strands				Total	Non-Interlock Strands			Total	Total
		Strand 2	Strand 3	Strand 5	Strand 6	Interlock Strands	Strand 1	Strand 4	Strand 7	Non-Interlock Strands	All Strands
1	Trp, Ile, Phe, Leu, Cys, Val, Met, Ala, Tyr	≥3	≥3	≥1	≥2	≥10 and 18≤	≥1 and 4≤	≥1 and 4≤	≥1 and 4≤	≥5 and 9≤	≥17 and 26≤
2	Ala	1≤	1≤	2≤	1≤	3≤	2≤	2≤	2≤	3≤	≥0 and 4≤
3	Phe, Trp, Tyr	2≤	3≤	2≤	2≤	6≤	2≤	1≤	2≤	3≤	≥2 and 8≤
4	Gln, Glu, Arg, Thr, Tyr, Asp, His, Lys, Ser, Asn	≥1	≥1	≥1	≥1	≥8 and 15≤	≥2 and 5≤	≥2 and 5≤	≥2 and 5≤	≥9 and 13≤	≥18 and 27≤
5	Glu, Arg, Lys, Asp, His	2≤	3≤	2≤	2≤	≥1 and 7≤	3≤	4≤	4≤	≥1 and 7≤	≥1 and 12≤
6	Pro, Gly, Asn, Asp, Glu	1≤	3≤	2≤	2≤	5≤	2≤	4≤	4≤	≥2 and 6≤	≥2 and 10≤
7	Pro	0	0	1≤	0	1≤	2≤	2≤	1≤	≥0 and 3≤	≥0 and 3≤
8	Gly	1≤	1≤	1≤	1≤	2≤	1≤	1≤	1≤	≥0 and 1≤	≥0 and 2≤
9	Pro + Gly + Ala	2≤	1≤	2≤	1≤	4≤	3≤	2≤	2≤	≥1 and 5≤	≥0 and 6≤

**Table 4:** Tests of specificity and sensitivity.

	Target	Other Proteins				
Rules	Proteins (144)	Beta-Proteins (3951)	Alpha-Proteins (3006)	c-Protein (3925)	d-Protein (3950)	Total (14,832)
1 All rules	130 (90%)	91 (2%)	3 (0.1%)	33 (0.8%)	21 (0.5%)	148 (0.9%)
2 The rules of Section 3.3	136 (94%)	2120 (54%)	442 (15%)	2259 (58%)	1369 (35%)	6190 (41.7%)
3 The rules of Section 3.2 and Section 3.3	134 (93%)	1653 (42%)	223 (7%)	1292 (33%)	808 (20%)	3976 (26.8%)
4 The rules of Section 3.1 and Section 3.3	130 (90%)	132 (3%)	6 (0.2%)	50 (1%)	34 (0.8%)	222 (2.4%)
5 The rules of Section 3.1	130 (90%)	796 (20%)	100 (3%)	896 (23%)	432 (11%)	2224 (14.9%)
6 All rules, exception rule 1a in Section 3.1	0	276 (7%)	15 (0.5%)	128 (3%)	71 (2%)	490 (3%)

## 4. Testing Specificity and Sensitivity of the “Rule Set” for Target Proteins

The rules contained in Section 3.1, Section 3.2, Section 3.3 and Section 3.4 constitute a complete set of rules that govern the distribution of favorable and unfavorable residues in target proteins. The rules’ specificity and sensitivity were tested on proteins in ASTRAL database. The sequences of 14,976 proteins that share less than 95% of sequence identity were divided into five subsets. In the first subset, there were 144 sequences of known target beta-sandwich proteins, while the other four subsets comprise all other proteins from different protein classes (Table 4).

Applying the rule set to the subset of target proteins yields 90% true positive sequences (row 1). Applying the rule set to “other” proteins gives very high specificity, only 148 out of 14,832 non-target proteins satisfied the rules of residue distribution for target proteins (row 1). Most of the false positives—91 out of 148—were beta proteins with sandwich-like structures, but with a different arrangements of strands. Importantly, almost none of the false positives had alpha helical structure. Thus, our rule set reliably differentiated beta sandwiches from alpha proteins. These results support our main hypothesis: knowledge of the distribution of a limited number of residues within sequences of a specified fold, formalized as a “rule set”, is sufficient for identification of protein structures of that fold with a high degree of sensitivity and specificity.

A distinct advantage of our approach is that it allows us to estimate the relative importance of specific residues for structure formation. To do so, we calculate the effect of removing one or several rules from the rule set on false positive rate. If removal of the particular rule results in a drastic increase of false positives, than it can be assumed that the particular rule encodes an essential sequence characteristic of target proteins.

Table 4 shows how omitting rules from the rule set affects specificity. This analysis reveals a kind of hierarchy among rules—some rules are more essential for structure prediction than others.

Row 1. The complete rule set is used for identification of target proteins.

Row 2. Only rules from Section 3.3 are used for identification of target proteins. It is shown that taking into account only distribution within strands and disregarding the rules describing the distribution of residues at hydrophobic and hydrophilic positions outside the strands dramatically increases false positive rate.

Row 3. Only rules from two Section 3.2 and Section 3.3 are used for identification of target proteins. Calculations showed that the addition of the rules for the distribution of residues in hydrophilic positions in strands does not significantly reduce the number of false positive sequences compared to Row 2 where only Section 3.3 rules were used.

Row 4. Only rules from Section 3.1 and Section 3.3 are used for identification of target proteins. In contrast to Rows 2 and 3, this variant, which takes into account distribution of residues at hydrophobic positions together with the distribution of residues within strands drastically decreased false positive rate. This analysis confirm the dominant role of hydrophobic core formation for protein architecture.

Row 5. Specifying residue content at hydrophobic positions in strands only is insufficient for identifying target proteins.

Row 6. We tested the necessity of requiring that three hydrophobic positions in interlock strands 2 and 3 be occupied by hydrophobic residues only (rule 1a in Section 3.1). In this test, all rules outlined in Section 3.1, Section 3.2, Section 3.3 and Section 3.4 are observed. We restricted only the number of hydrophobic residues in strands 2 and 3 to no more than 2. The total number of hydrophobic residues in four interlock strands, however, was kept the same at no less than nine residues. Results of the calculation showed that “redistribution” of hydrophobic residues in interlock strands significantly increases the false positive rate. This demonstrates that the requirement of three hydrophobic residues in strands 2 and 3 is more important for the target protein fold than some of the other rules. This finding suggests a hierarchy among four interlock strands. The first two strands, strands 2 and 3, play a predominant role in the interlock and are essential to formation of the hydrophobic core.

## 5. Discussion

Whether reliable sequence/structure correlation analysis can be made by traditional methods of sequence analysis is uncertain. In the widely used sequence alignment procedures, such as BLAST and EMBOSS, the reliability of conserved positions detection strongly depends on the degree of similarity of analyzed sequences. For example, EMBOSS test revealed 9.6% identity and 13.6% similarity of residues in two target proteins with the same protein fold. The pairwise alignment does not correctly identify the secondary structures in query proteins. BLAST method applied to these two sequences yields “no significant similarity”.

Using methods of hidden Markov models to develop sequence rules may also be problematic, mostly because “a hit” of these methods depends on the statistical profile of HMM, which is based on consensus of multiple sequence alignment. HMM works well for large protein families, such as Ig proteins, for which there exist reliable statistics of residue frequency at each position. HHpred method correctly detected Ig fold for all target Ig-like proteins in our test. To more accurately estimate prediction potential of HHM, it would be necessary to test this method on a set of limited number of proteins with the same fold, but widely diverse sequences for which multiple sequence alignment is not obvious. The result of multiple sequence alignment for these dissimilar sequences may be ambiguous.

Our work demonstrates the possibility of developing an alternative approach to predicting structure from sequence based on formulation of rules that capture the essential aspects of the sequence–structure relationship for a particular fold. Our hypothesis is that general principles of protein structure formation translate into fold-specific constraints at a sequence level. For example, the most significant criterion for protein stability is the requirement of a hydrophobic core. To understand the constraints that this criterion imposes on target proteins, we ask detailed questions: how many hydrophobic residues are necessary to form a stable core? How are the hydrophobic residues to be distributed in a sequence of the given fold? Another example is the well-known limitation on the number of Pro and Gly residues allowed in beta strands. In our analysis, we determine the constraints on Pro and Gly residues in different strands of target proteins.

The most important step in our strategy is to make assumptions about arrangement of key residues in a subset of sequences of structurally similar proteins. The initial assumptions are tested on target proteins and then fine-tuned, based on the data fit. This iterative testing allows us to accept the assumption as the “rule” for target proteins, if it fits well with the data, or to further relax the assumption, or to make it more specific, or to reject the assumption.

It is important to note here that in our approach unlike HMM methods it is not necessary to know residue content at every, or even most, positions of the chain to make a structure prediction. Moreover, our approach does not even require precise localization of key positions in a sequence; an approximate localization is often sufficient. For example, one of the rules 1a in Section 3.1 states that at least one unspecified position within a fragment, which correspond to a strand, should be occupied by hydrophobic residues. Another example is the rules for loops (Section 3.4).

Another significant difference between HMM method and the current approach is that HMM is essentially a probabilistic algorithm, whereas in our approach no probabilistic information is required for structure prediction. We ask focused questions about residue content such as: Does a particular strand contain a hydrophobic residue in any of selected positions in all structures with a given fold? If the answer is “Yes”, this condition is formulated as the rule. Thus, our approach is based not on probabilities of finding certain residues at certain positions, but on checking a query sequence against a set of rules specific for a particular fold. Conceptually and computationally, the proposed approach is different from HHM methods.

Our objective is to find a sufficient number of rules for a certain supersecondary structure—“the rule set”—that is specific and sensitive. The possibility of finding such a rule set for target proteins in this research demonstrates that the distribution of a limited number of favorable and unfavorable residues in sequences, rather than overall homology, defines the protein structure.

Our assumptions are based on the general principle of structure formation, but because the sequence/structural rules have been tested on existing structures, a potential drawback of our approach is its dependence on the content of the structural protein database. The important question is therefore how general is this approach. It was shown that the set of rules is highly sensitive (~90%) and very specific (~99%) for identifying sequences of proteins. Thus, this method reliably distinguishes proteins with the given fold from other proteins. Based on these data it may be supposed that it is unlikely that the number of false positive proteins will increase in the analysis of other sequence databases, but there is some possibility that this approach will not be able to find some of the true positive proteins.

An important advantage of the “rule-based” approach over the homology-based ones is that it allows one to “weigh” the relative structural importance of residues in the various positions of strands. We found that the most critical characteristics for the target proteins relate to the distribution of residues in strands 2 and 3. This observation may be relevant to understanding of the dynamics of protein structure formation. It could be suggested that, in the initial stages of folding, two neighboring fragments in the beginning of a polypeptide chain form contacts with each other. The result of this interaction is the creation of two strands-strands 2 and 3, which initiate beta sheet formation. Further development of structure and formation of other strands leads to separation of strands 2 and 3 in space. We speculate that foremost among the various factors that drive rearrangement of strands to form a two beta sheet structure is the irregular distribution of hydrophobic residues in a sequence. About half of all hydrophobic residues are located in strands 2 and 3, and the rest are distributed more-or-less uniformly among the remaining strands. If strands 2 and 3 were to be localized on one beta sheet, this would prevent uniform distribution of inter-residue hydrophobic contacts between two beta sheets, and thereby disrupting formation of a stable hydrophobic core. In target proteins, the difference between the numbers of hydrophobic residues in the two sheets that form the hydrophobic interior is usually no more than two. Since strands 2 and 3 have the largest number of hydrophobic residues, their localization in the two different beta sheets allows for maximum number of hydrophobic contacts between two sheets. According to our hypothesis, the distribution of hydrophobic residues in strands largely defines the specific beta fold. Thus, beta-sandwich and beta-barrel structures may be distinguished by the different distributions of hydrophobic residues involved in hydrophobic core formation. Certainly, other factors may also be important for strand arrangement and contribute to structure stability as well. The specific sequence rules required for the strand arrangement that characterizes sandwich proteins are listed in Section 3.

## 6. Conclusions

Protein sequences, as any non-random and meaningful “text”, obey certain rules of distribution of residues within polypeptide chains. These “grammatical” rules ensure the stability of a folded chain in 3D space or, more specifically, provide an adequate number of hydrophobic residues in structure’s interior and of hydrophilic residues at the surface. This important condition imposes restrictions on residue content at certain location in a sequence—so called “unfavorable” residues that are incompatible with the given fold. The present research shows how the knowledge of main structural features of a specific group of protein structures allows one to deduce main rules of residue distribution for these proteins.

The proposed method was designed to answer the following questions: how the structural features of the given fold-number of strands, an arrangement of strands in beta sheets, formation of two beta-sheets,* etc.* are “projected” onto a sequence, and how does the distribution of key residues in sequences determine the folding of a polypeptide chain. Knowledge of the sequence constraints allows us to make meaningful structure predictions for query sequences independent of their degree of similarity with known protein structures.

This approach may also prove useful for solving the inverse problem of the sequence–structure relationship: deducing crucial sequence features from structure. The knowledge of which residues are favorable and where they should be located in a chain in order to form a particular fold could prove useful for rational protein engineering design as well for predicting the effect of mutations on structure.
